# Initiation of decohesion between a flat punch and a thin bonded incompressible layer

**DOI:** 10.1177/10812865241240484

**Published:** 2024-05-06

**Authors:** Ivan I Argatov, Gennady S Mishuris, Valentin L Popov

**Affiliations:** College of Aerospace Engineering, Chongqing University, Chongqing, China; Institut für Mechanik, Technische Universität Berlin, Berlin, Germany; Department of Mathematics, Aberystwyth University, Aberystwyth, UK; Institut für Mechanik, Technische Universität Berlin, Berlin, Germany; National Research Tomsk State University, Tomsk, Russia

**Keywords:** Adhesive contact, JKR-type model, perturbation, elliptical punch, incompressible layer

## Abstract

Non-axisymmetric frictionless JKR-type adhesive contact between a rigid body and a thin incompressible elastic layer bonded to a rigid base is considered in the framework of the leading-order asymptotic model, which has the form of an overdetermined boundary value problem. Based on the first-order perturbation of the Neumann operator in the Dirichlet problem for Poisson’s equation, the decohesion initiation problem is formulated in the form of a variational inequality. The asymptotic model assumes that the contact zone and its boundary contour during the detachment process are unknown. The absence of the solvability theorem is illustrated by an example of the instability of an axisymmetric flat circular contact.

## 1. Introduction

Adhesion between surfaces of contacting solids is usually observed as a reversible phenomenon [1] and is widely used in practice for temporary connecting flat parts [2]. An often encountered situation in engineering is when one of the contacting solids can be regarded as absolutely rigid (e.g., made of steel) whereas another one is fabricated from a highly adhesive thin layer of rubber-like material. Namely, this combination was utilized in recently conducted experiments in Lyashenko and Popov [3] and Li et al. [4]. In particular, it was noticed that the theory of adhesive contact created by Johnson et al. [5] is suitable for fitting quasi-static decohesion processes.

In the present mathematical modeling study, we develop an asymptotic model for describing the quasi-static adhesive detachment process, which is characterized by a small variation of the contact zone. Our asymptotic model of the JKR-type adhesive contact [6] for a thin incompressible elastic layer [7] serves as a cornerstone of the developed decohesion model. We note that different contact problems (with a known or an a priori unknown contact zone) for a thin incompressible elastic layer were previously examined in the literature [8–11] (see [12] for more references) using different analytical techniques. The JKR-type adhesive contact for an incompressible layer was previously considered in Yang [13], but only in the axisymmetric setting. Here, the main difficulty is the absence of this simplifying assumption.

Recently, a perturbation study of the JKR model was conducted in Argatov [14] in the basic case of local contact when the Hertzian elastic half-space hypothesis applies. Using a direct analogy with crack propagation [15–17] (which for the JKR-type contact has been established in Maugis [18, 19]; see also [20]), the variational equation for a contact contour perturbation was obtained in Argatov [14, 21]. In the present work, an additional difficulty is provided by the assumption that the contact contour may be partially perturbed (as in the crack propagation problem [17, 22]), as it is the case in the stage of decohesion initiation.

It should be emphasized that a concept of a partially perturbed contour has been previously utilized in asymptotic modeling of crack propagation [17]. Here, we make use of this analogy with fracture mechanics [23] and derive a variational inequality for the contact contour perturbation similar to the original studies [17, 22]. Still, this study is limited to the phenomenon of decohesion initiation as a flat punch contact geometry is assumed. In contrast to the original study by Kendall [24], we consider an arbitrary flat punch (with a smoothly parameterized edge contour) being in a full JKR-type adhesive contact with a thin incompressible elastic layer instead of a thin compressible elastic layer bonded to a rigid base (see section 5 “Strength of glue films”). Although the assumption of incompressibility drastically changes the thin layer’s elastic response, the energy balance considerations in the axisymmetric case of a cylindrical punch support Kendall’s finding that as fracture of the adhesive joint proceeds, a surplus energy is produced with a meaning that the initiated decohesion process is unstable (appearing as an acceleration of a propagating crack).

The rest of this paper is organized as follows. First, we formulate the problem of a quasi-static decohesion initiation in the asymptotic modeling framework [7]. Then, following [25, 26], we derive the first-order perturbation formula for the Neumann operator in the Dirichlet problem for Poisson’s equation. After that, following [22], we outline a variational formulation for the decohesion initiation problem. The absence of the solvability theorem is illustrated by two examples, including one about the instability of a flat circular adhesive contact. Finally, we outline a discussion of the obtained results and formulate concluding remarks.

## 2. Asymptotic model of the JKR-type adhesive contact

**Figure 1.**
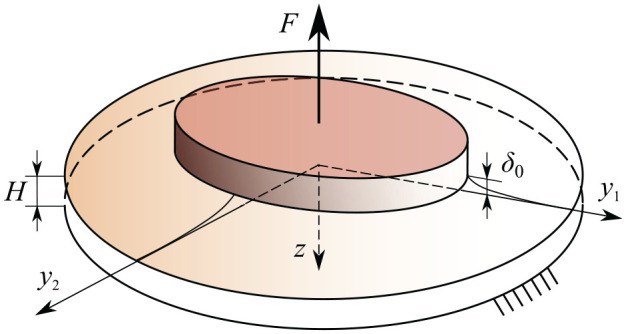
Schematics of the adhesive detachment of a flat-ended punch from a thin elastic layer bonded to a rigid base.

We consider an incompressible transversely isotropic elastic layer bonded to a flat rigid base (see Figure 1). Let H denote the layer thickness. The elastic layer is referred to a Cartesian frame with the in-plane coordinates denoted by $y_{1}$ and $y_{2}$ . We assume that the vertical z -axis is pointed inside the elastic layer such that the planes z=0 and z=H represent the upper (free) and bottom (fixed) surfaces of the layer in the undeformed state.

The upper surface of the elastic layer can experience an adhesive contact with a rigid solid (henceforth called the punch), which is characterized by the shape function φ(y) , such that φ(0)=0 and φ(y)≥0 . By definition of the shape function, the equation z=−φ(y) describes the punch surface that can contact the layer, thereby inducing some deformed state in the elastic layer. Owing to the indicated properties of the shape function, the initial contact between the punch and the elastic layer occurs at least at one point, including the center of coordinates. In the special case of a flat-ended punch, we have φ(y)≡0 .

The level of contact stresses produced in the elastic layer in the deformed state can be characterized either by the value of external load, F , pressing the punch against the layer’s upper surface, or by the value of the punch vertical displacement, $\delta_{0}$ , both of which are positive in the loading stage when the punch approaches the rigid base.

Under the simplifying assumption that friction forces at the contact interface may be neglected, the frictionless contact between the punch and the elastic layer can be characterized by the density p(y) of contact pressures distributed over a contact zone ω . The equation of static equilibrium implies that:

(1) $$F=\int \int_{\omega }p(y)dy.$$

By exploiting the assumption that the characteristic sizes of the contact domain ω are much larger than the layer thickness H , the following asymptotic model has been established [12]:

(2) $$-\frac{H^{3}}{3G^{\prime }}\Delta_{y}p(y)=\delta_{0}-\varphi (y),\;y\in \omega ,$$

(3) p(y)=0,y∈Γ,

here, $\Delta_{y}=\partial^{2}/\partial y_{1}^{2}+\partial^{2}/\partial y_{2}^{2}$ is the Laplacian, Γ is the contact contour, i.e., Γ=∂ω , and $G^{\prime }$ is the out-of-plane shear elastic modulus of the incompressible elastic layer.

In the case of unilateral contact, both the contact domain ω and its boundary Γ are a priori unknown and an additional boundary condition should be introduced. In particular, if the contact interaction is assumed to be non-adhesive, then, in order to fix this ambiguity, the Neumann boundary condition ∂p(y)/∂n=0 should be imposed on Γ (see [8, 9, 12] for details). In what follows, we assume that the contact between the elastic layer and the punch is adhesive of the JKR type and impose the following additional boundary condition [7]:

(4) $$\frac{\partial p}{\partial n}(y)=-\frac{1}{H^{3/2}}\sqrt{6G^{\prime }\Delta \gamma },\;y\in \Gamma ,$$

here, ∂/∂n is the *inward* normal derivative, and Δγ is the surface energy density.

We note that the boundary-value problem (2)–(4) is known as the Cauchy problem for Poisson’s equation and is severely ill-posed [27]. However, it should be remembered that the contour Γ is not fixed and the contact problem (2)–(4) can be classified as a free boundary problem [28].

### 2.1. Problem of decohesion initiation

To fix our ideas, we consider the case of a flat-ended punch, when φ(y)≡0 and the initial contact is realized over a *given* domain ω . Generally speaking, besides a vertical translational displacement $\delta_{0}$ , the punch can be tilted during loading by rotating around the horizontal axes to some small angles $\beta_{1}$ and $\beta_{2}$ . So, in the loading stage, the contact pressure density p(y) solves the Dirichlet problem:

(5) $$-\frac{H^{3}}{3G^{\prime }}\Delta_{y}p(y)=\delta_{0}-\beta_{2}y_{1}+\beta_{1}y_{2},\;y\in \omega ,$$

(6) p(y)=0,y∈Γ.

According to our previous analysis [7], the condition of full JKR-type contact can be formulated as:

(7) $$-\frac{\partial p}{\partial n}(y)\leq \frac{1}{H^{3/2}}\sqrt{6G^{\prime }\Delta \gamma },\;y\in \Gamma .$$

In other words, so long the above condition is satisfied, the contact between the flat-ended punch and the surface of the thin incompressible elastic layer is maintained over the entire domain ω .

The decohesion starts at a point or several points where the inequality (7) turns into equality. We assume that after the decohesion initiation, the process of quasi-static detachment, which can be characterized by a time-like variable τ , will produce a contact zone $\omega_{\tau }$ with a partially unknown contour $\Gamma_{\tau }$ , being a subdomain of ω . To describe the commencing stage of detachment, when the contact zone $\omega_{\tau }$ slightly differs from ω , we will treat the partially unknown contact contour $\Gamma_{\tau }$ as a perturbation of the initial contact contour Γ .

**Figure 2.**
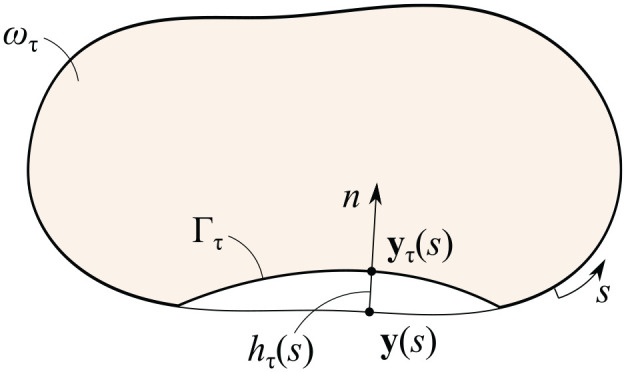
Perturbation of the contact domain.

With this aim, we introduce the natural parametrization of the contour Γ , using the arc-length variable s∈[0,l) , where l is the length of the curve Γ . It is assumed that the domain ω is simply connected and that the positive direction of the local coordinate s is anti-clockwise (see Figure 2).

Let t(s) and n(s) be the unit tangential and *inward* (with respect to the domain ω ) normal vectors to the contour Γ , respectively. We assume that the partially unknown contour $\Gamma_{\tau }$ can be described in the local coordinates (s,n) by the equation:

(8) $$n=h_{\tau }(s),\;s\in [0,l),$$

where n is the distance measured along the inward normal to the contour Γ .

Thus, if y(s) denotes the point on the contour Γ , which corresponds to the parameter s , then equation (8) determines the point $y_{\tau }(s)=y(s)+h_{\tau }(s)n(s)$ on the contour $\Gamma_{\tau }$ .

The function $h_{\tau }(s)$ should be determined by satisfying the conditions:

(9) $$h_{\tau }(s)>0\;\;\Rightarrow \;\;N_{\tau }=N_{c},$$

(10) $$N_{\tau }<N_{c}\;\;\Rightarrow \;\;h_{\tau }(s)=0.$$

Here, we have introduced the notation:

(11) $$N_{\tau }(s)=-\frac{\partial p_{\tau }}{\partial n}(y_{\tau }(s)),\;y_{\tau }(s)\in \Gamma_{\tau },$$

(12) $$N_{c}=\frac{1}{H^{3/2}}\sqrt{6G^{\prime }\Delta \gamma }.$$

Furthermore, it can be shown that the unit inward normal vector to the curve $\Gamma_{\tau }$ can be evaluated as:

(13) $$n_{\tau }(s)=\frac{\left(1+\kappa_{\tau }(s)h_{\tau }(s))n(s)-h_{\tau }^{\prime }(s)t(s\right)}{\sqrt{{\left(1+\kappa_{\tau }(s)h_{\tau }(s)\right)}^{2}+h_{\tau }^{\prime 2}(s)}},$$

where $\kappa_{\tau }(s)$ is the curvature of the contour $\Gamma_{\tau }$ , and the prime denotes the derivative with respect to the variable s .

For a slightly perturbed contact contour, both functions $h_{\tau }(s)$ and $h_{\tau }^{\prime }(s)$ can be assumed to be small, and therefore, equation (13) implies that $n_{\tau }(s)≃n(s)$ , or to be more precise:

(14) $$n_{\tau }(s)≃n(s)-h_{\tau }^{\prime }(s)t(s),$$

when the terms of the second order of smallness have been neglected.

Now, let $\nabla_{y}$ denote the gradient operator $\left(\partial /\partial y_{1},\partial /\partial y_{2}\right)$ . Then, by definition (see also equation (11)), we have:

(15) $$N_{\tau }(s)=-n_{\tau }(s)\cdot \nabla_{y}p_{\tau }(y){|}_{y=y_{\tau }(s)},$$

where the dot denotes the scalar product.

Finally, we note that so far the dependence of the contact pressure density $p_{\tau }(y)$ on the time-like variable τ was not discussed for the sake of simplicity. This functional dependence comes through the dependence of the kinematic coefficients on the right-hand side of equation (5). In what follows, we consider the case of translational (without tilting) detachment, when equation (5) is replaced with the following one:

(16) $$-\frac{1}{m}\Delta_{y}p_{\tau }(y)=\delta_{0}+\tau \delta_{1},\;y\in \omega_{\tau },$$

here, $\delta_{0}$ and $\delta_{1}$ are given constants, and we have introduced the notation:

(17) $$m=\frac{3G^{\prime }}{H^{3}}.$$

Without any loss of generality, we may fix the constant $\delta_{0}$ in such a way that the solution $p_{0}(y)$ , $y\in \omega_{0}=\omega$ , corresponds to the critical state when the maximum of the *outward* normal derivative $N_{0}(s)$ coincides with the critical value $N_{c}$ defined by formula (12). Obviously, in the special case of a circular flat-ended punch, the critical state in the translational detachment is reached when $N_{0}(s)\equiv N_{c}$ , i.e., the decohesion should be initiated along the entire circular contour Γ . The time-like variable τ is assumed to be counted from the moment of critical state ( τ=0 ), and the constant $\delta_{1}$ represents the detachment rate.

Thus, the concrete problem of decohesion initiation, which will be studied in the next sections, is comprised of equation (16), relations (9), (10), and the Dirichlet boundary condition

(18) $$p_{\tau }(y_{\tau }(s))=0,\;y_{\tau }(s)\in \Gamma_{\tau }.$$

In particular, we are interested in the special case of an elliptical domain of initial contact, which is consistent with the experimental setup utilized in Li et al. [4].

## 3. Perturbation of the Neumann operator

By treating the variable τ as a perturbation parameter, we consider the limit case ( τ=0 ) that is formed by the relations:

(19) $$-\frac{1}{m}\Delta_{y}p_{0}(y)=\delta_{0},\;y\in \omega ,$$

(20) $$p_{0}(y(s))=0,\;y(s)\in \Gamma ,$$

(21) $$N_{\tau }\leq N_{c},\;s\in [0,l),$$

which follow from relations (16), (18), and (7), respectively.

The Neumann operator is defined as follows (see equation (15)):

(22) $$N_{0}(s)=-n(s)\cdot \nabla_{y}p_{0}(y){|}_{y=y(s)}.$$

Now, abstracting from relation (21) and considering the Dirichlet boundary-value problems (19) and (20), let us examine the perturbation of the Neumann operator under a small perturbation of the boundary Γ , as it is described by equation (8). In other words, we consider the perturbed Dirichlet problem:

(23) $$-\frac{1}{m}\Delta_{y}p_{\tau }(y)=\delta_{0},\;y\in \omega_{\tau },$$

(24) $$p_{\tau }(y_{\tau }(s))=0,\;y_{\tau }(s)\in \Gamma_{\tau }.$$

We note that equation (23) differs from equation (16) by dropping out the driving term $\tau \delta_{1}$ , as we are interested in separating out the effect of the contour perturbation.

To simplify the analysis, we put:

(25) $$h_{\tau }(s)=\tau h(s),$$

where h(s) is assumed to be independent of the parameter τ .

Following Argatov and Mishuris [12], we employ the perturbation technique (see, e.g., [29]) and formally write:

(26) $$p_{\tau }{|}_{\Gamma_{\tau }}=p_{\tau }{|}_{\Gamma_{0}}{+\tau h\frac{\partial p_{\tau }}{\partial n}|}_{\Gamma_{0}}+O(\tau^{2}),$$

where $\Gamma_{0}=\Gamma$ is the unperturbed contour.

In light of the first-order expansion (26), we look for the solution to the perturbed problems (23) and (24) in the form:

(27) $$p_{\tau }(y)=p_{0}(y)+\tau {\tilde{p}}_{1}(y)+\ldots$$

The substitution of the asymptotic expansions (26) and (27) into the boundary condition (24), in view of equation (20), yields the non-homogeneous Dirichlet boundary condition:

(28) $${\tilde{p}}_{1}(y(s))=-h(s)\frac{\partial p_{0}}{\partial n}(y(s)),\;y(s)\in \Gamma .$$

Moreover, the substitution of equation (27) into equation (23), in view of equation (19), yields the Laplace equation:

(29) $$\Delta_{y}{\tilde{p}}_{1}(y)=0,\;y\in \omega .$$

We also note that, in view of equation (22), equation (28) can be rewritten as:

(30) $${\tilde{p}}_{1}(y(s))=h(s)N_{0}(s),\;y(s)\in \Gamma .$$

Now, in view of the first-order approximation (14), for the unit normal vector $n_{\tau }(s)$ , the Neumann operator (15) can be approximated as:

$$N_{\tau }(s)≃(-n(s)\cdot \nabla_{y}p_{\tau }(y)+\tau h^{\prime }(s)t(s)\cdot \nabla_{y}p_{\tau }(y)){|}_{y=y(s)},$$

which can be further simplified by making use of the asymptotic expansion (27) as:

$$N_{\tau }(s)≃-\frac{\partial p_{0}}{\partial n}(y)-\tau h(s)\frac{\partial^{2}p_{0}}{\partial n^{2}}(y)-\tau \frac{\partial {\tilde{p}}_{1}}{\partial n}(y)+\tau h^{\prime }(s)\frac{\partial p_{0}}{\partial s}(y){|}_{y=y(s)}.$$

Finally, taking into account equations (20) and (22), we simplify the above asymptotic formula as follows:

(31) $$N_{\tau }(s)≃N_{0}(s)-\tau \frac{\partial {\tilde{p}}_{1}}{\partial n}(y(s))-h_{\tau }(s)\frac{\partial^{2}p_{0}}{\partial n^{2}}(y(s)),$$

where y(s)∈Γ for s∈[0,l) .

To this end, we still need to relate the normal derivative $\partial {\tilde{p}}_{1}/\partial n$ to the boundary values of the function ${\tilde{p}}_{1}(y)$ as they are specified by the boundary condition (30). With this aim, we introduce the Steklov–Poincaré (Dirichlet-to-Neumann) operator $S:H^{1/2}(\Gamma )\to H^{-1/2}(\Gamma )$ as:

(32) $$(Sg)(s)=-\frac{\partial u}{\partial n}(y(s)),\;y(s)\in \Gamma ,$$

where u(y) is the solution of the Dirichlet problem:

$$\Delta_{y}u(y)=0,\;y\in \omega ;\;u(y(s))=g(s),\;y(s)\in \Gamma .$$

We note that by the application of Green’s identity, it can be easily established that:

(33) $$\int \int_{\omega }|\nabla_{y}u(y){|}^{2}dy=\int_{\Gamma }g(s)(Sg)(s)ds.$$

Thus, in view of equations (25), (30), and (32), formula (31) can be represented in the form:

(34) $$N_{\tau }(s)≃N_{0}(s)+(Sh_{\tau }N_{0})(s)-h_{\tau }(s)\frac{\partial^{2}p_{0}}{\partial n^{2}}(y(s)).$$

We note that:

$$\Delta_{y}p_{0}(y){|}_{y\in \Gamma }=\frac{\partial^{2}p_{0}}{\partial n^{2}}(y(s))-\kappa (s)\frac{\partial p_{0}}{\partial n}(y(s)),$$

and thus, in view of equations (19) and (22), we have:

(35) $$\frac{\partial^{2}p_{0}}{\partial n^{2}}(y(s))=-\kappa (s)N_{0}(s)-m\delta_{0},$$

where κ(s) is the curvature of the unperturbed contour Γ , and the constant m is defined by equation (17).

The first-order perturbation formula (34) will be used in formulating the problem of decohesion initiation.

## 4. Variational formulation of the decohesion initiation problem

Let us return to the problems (16), (18), and (9)–(11). By comparing equations (16) and (19), we readily see that an additional term should be introduced to the right-hand side of formula (34) as:

(36) $$\begin{array}{c} N_{\tau }(s)≃N_{0}(s)+\tau N_{1}(s)+(Sh_{\tau }N_{0})(s)\\ \;\;\;\;\;\;\;\;\;\;\;\;-h_{\tau }(s)[\kappa (s)N_{0}(s)+m\delta_{0}], \end{array}$$

where

(37) $$N_{1}(s)=-n(s)\cdot \nabla_{y}p_{1}(y){|}_{y=y(s)},$$

and $p_{1}(y)$ is the solution of the Dirichlet problem:

(38) $$-\frac{1}{m}\Delta_{y}p_{1}(y)=\delta_{1},\;y\in \omega ;\;p_{1}(y(s))=0,\;y(s)\in \Gamma .$$

Furthermore, in view of equations (7) and (9)–(11), we have:

(39) $$N_{\tau }(s)\leq N_{c},$$

(40) $$h_{\tau }(s)>0\;\;\Rightarrow \;\;N_{\tau }(s)=N_{c},$$

(41) $$h_{\tau }(s)=0\;\;\Rightarrow \;\;N_{\tau }(s)\leq N_{c},$$

(42) $$h_{\tau }(s)\geq 0,\;s\in [0,l).$$

From equations (40)–(42), it follows that $[N_{c}-N_{\tau }(s)]h_{\tau }(s)=0$ for every s∈[0,l) , so that:

(43) $$\int_{\Gamma }[N_{c}-N_{\tau }(s)]h_{\tau }(s)ds=0.$$

Let χ(s) denote a trial function such that $\chi \in C^{\infty }(\Gamma )$ and χ(s)≥0 on [0,l) . Then, in view of equations (40) and (41), we can write $[N_{c}-N_{\tau }(s)]\chi (s)\geq 0$ . However, since the Steklov–Poincaré operator S in equation (36) acts on the function $h_{\tau }(s)N_{0}(s)$ , we need the inequality $[N_{c}-N_{\tau }(s)]\chi (s)N_{0}(s)\geq 0$ , which is a consequence of relations (40) and (41), provided $N_{0}(s)\geq 0$ . At the same time, in the case of detachment loading when $\delta_{0}<0$ , the strong maximum principle [30] applies.

Therefore, for any trial function χ(s) , we obtain:

(44) $$\int_{\Gamma }[N_{c}-N_{\tau }(s)]N_{0}(s)\chi (s)ds\geq 0.$$

Thus, in the framework of the general scheme [31], from equations (43) and (44), it follows that:

(45) $$(N_{c}-N_{\tau },N_{0}\chi -N_{0}h_{\tau }{)}_{\Gamma }\geq 0\;\forall \chi \in C^{\infty }(\Gamma ),\;\;\chi \geq 0,$$

where $(\cdot ,\cdot {)}_{\Gamma }$ denotes the inner product in $L_{2}(\Gamma )$ , i.e.,

$$(f,g{)}_{\Gamma }=\int_{\Gamma }f(s)g(s)ds.$$

Observe that the variational inequality (45) is of the same type as those studied in Kolton and Nazarov [22]. Following Kolton and Nazarov [22], we introduce the notation:

(46) $$H=\frac{N_{0}}{N_{c}}h_{\tau },\;f_{0}=\frac{N_{0}}{N_{c}}-1,\;f_{1}=\frac{N_{1}}{N_{c}},\;b=-\kappa -\frac{m\delta_{0}}{N_{0}}.$$

Then, the substitution of equation (36) into equation (45) yields:

(47) $$(\mathrm{bH},X-H{)}_{\Gamma }-(SH,X-H{)}_{\Gamma }\geq (f_{0}+\tau f_{1},X-H{)}_{\Gamma },$$

for all $X\in C^{\infty }(\Gamma )$ such that X(s)≥0 , s∈[0,l) .

The main problem with the variation inequality (47) is that even in the case when b(s)>0 for every s∈[0,l) , the symmetric quadratic form $(\mathrm{bH},H{)}_{\Gamma }-(SH,H{)}_{\Gamma }$ is not positively defined, because $(SH,H{)}_{\Gamma }\geq 0$ according to the identity (33). Hence, the operator −S+b is not $H^{1/2}$ -coercive, and therefore, the solvability of the variational inequality (47) cannot be established from general theorems.

## 5. Examples

In this section, we consider two special cases of the contact geometry, which are of interest for interpreting the experimental results obtained in Li et al. [4].

### 5.1. Elliptical contact domain

We consider the Dirichlet problems (19) and (20) in an elliptical domain ω bounded by an ellipse Γ , which is described by the canonical equation:

(48) $$\frac{y_{1}^{2}}{a^{2}}+\frac{y_{2}^{2}}{b^{2}}=1,$$

where without loss of generality, we assume that a≥b . It is convenient to make use of the parametric representation $y_{1}=a\cos\theta$ , $y_{2}=b\sin\theta$ for θ∈[0,2π) . In such a case, the unit vector of inner normal at a point y(s)∈Γ is:

(49) $$n(\theta )=\frac{\left(-b\cos\theta ,-a\sin\theta \right)}{\sqrt{a^{2}\sin^{2}\theta +b^{2}\cos^{2}\theta }}.$$

It can be easily verified that the solution of the Dirichlet problems (19) and (20) is given by:

(50) $$p_{0}(y)=\frac{m\delta_{0}}{2}\frac{a^{2}b^{2}}{\left(a^{2}+b^{2}\right)}\left(1-\frac{y_{1}^{2}}{a^{2}}-\frac{y_{2}^{2}}{b^{2}}\right),$$

so that:

(51) $$\nabla_{y}p_{0}(y)=-m\delta_{0}\frac{a^{2}b^{2}}{\left(a^{2}+b^{2}\right)}\left(\frac{y_{1}}{a^{2}},\frac{y_{2}}{b^{2}}\right).$$

From equations (49) and (51), it follows that:

(52) $$\frac{\partial p_{0}}{\partial n}(\theta )=m\delta_{0}\frac{a^{2}b^{2}}{\left(a^{2}+b^{2}\right)}\sqrt{a^{2}\sin^{2}\theta +b^{2}\cos^{2}\theta }.$$

We recall that in the case of detachment, we have $\delta_{0}<0$ . So, by noticing that the maximum of the expression $\sqrt{a^{2}\sin^{2}\theta +b^{2}\cos^{2}\theta }$ is achieved at the points θ=π/2 and θ=3π/2 , we can state that the decohesion starts at the vertices of the *minor* axis (we recall that a>b ), provided the punch displacement $\delta_{0}$ satisfies the equation:

(53) $$-m\delta_{0}\frac{a^{2}b^{2}}{\left(a^{2}+b^{2}\right)}=N_{c},$$

where the positive parameter $N_{c}$ is defined by formula (12) in terms of the work of adhesion Δγ . This a priori unexpected finding has been previously established in Argatov and Popov [32].

Thus, in view of equations (22), (52), and (53), we can write:

(54) $$N_{0}(\theta )=\frac{N_{c}}{a}\sqrt{a^{2}\sin^{2}\theta +b^{2}\cos^{2}\theta }.$$

Furthermore, the curvature of the elliptical contour Γ can be evaluated as $\kappa (\theta )=\mathrm{ab}(a^{2}\sin^{2}\theta +b^{2}\cos^{2}\theta {)}^{-3/2}$ , and therefore, in view of equations $(46{)}_{4}$ , (53), and (54), we find that:

$$b(\theta )=\frac{a^{4}\sin^{2}\theta +b^{4}\cos^{2}\theta }{\mathrm{ab}{\left(a^{2}\sin^{2}\theta +b^{2}\cos^{2}\theta \right)}^{3/2}},$$

and it is readily seen that b(θ)>0 for every θ∈[0,2π) .

This example illustrates the negative assertion made above regarding the solvability of the variational inequality (47).

### 5.2. Instability of circular adhesive contact

Now, we consider a circular domain ω of radius a centered at the origin of coordinates, so that the initial contact contour Γ is described by the equation $y_{1}^{2}+y_{2}^{2}=a^{2}$ . In view of equations (50) and (53) applied for b=a , we have:

(55) $${\frac{\partial^{2}p_{0}}{\partial n^{2}}|}_{\Gamma }=\frac{N_{c}}{a}.$$

Let $\Gamma_{\tau }$ denote a small perturbation of Γ that preserves the circular shape. To be more specific, we assume that $\Gamma_{\tau }$ is a circle of radius $a-d_{\tau }$ with the center displaced to some small distance $d_{\tau }$ from the center of the circle Γ . So, to fix our stand-point, let $\Gamma_{\tau }$ be described by the equation $y_{1}^{2}+(y_{2}+d_{\tau }{)}^{2}=(a-s_{\tau }{)}^{2}$ .

Then, in the polar coordinates, we will have:

(56) $$r=a-h_{\tau }(\theta ),\;h_{\tau }(\theta )≃d_{\tau }(1+\sin\theta ).$$

In the case under consideration, the solution to the Dirichlet problems (19) and (20) is given by formula (50) with b=a , and thus, equation (54) immediately implies that:

(57) $$N_{0}(\theta )\equiv N_{c}.$$

Using Poisson’s formula, it can be shown that:

$$(Sg)(\theta )=\frac{1}{4\pi a}\int_{0}^{2\pi }\frac{\left(g(t)-g(\theta )\right)}{\sin^{2}[(t-\theta )/2]}dt,$$

where the integral is understood in the Cauchy principal value sense, and:

(58) $$S\left\{\begin{array}{c} \cos n\theta \\ \sin n\theta \end{array}\right\}=\frac{n}{a}\left\{\begin{array}{c} \cos n\theta \\ \sin n\theta \end{array}\right\},\;n=1,2,\ldots$$

Thus, according to equations $(56{)}_{2}$ , (57), and (58), we will have:

(59) $$(Sh_{\tau }N_{0})(\theta )=N_{c}\frac{d_{\tau }}{a}\sin\theta .$$

Since in the axisymmetric case, the decohesion initiates along the entire contour Γ , we impose the condition:

(60) $$N_{\tau }(\theta )=N_{c},\;\theta \in [0,2\pi ).$$

At the same time, the asymptotic formulas (34) and (36), in view of equations (55), $(56{)}_{2}$ , and (59), imply that:

(61) $$N_{\tau }(\theta )≃N_{c}+\tau N_{1}(\theta )+N_{c}\frac{d_{\tau }}{a}\sin\theta -N_{c}\frac{d_{\tau }}{a}\sin\theta (1+\sin\theta ).$$

Hence, the substitution of equation (61) into equation (60) yields:

(62) $$\tau N_{1}(\theta )=N_{c}\frac{d_{\tau }}{a}.$$

We recall that the Neumann operator $N_{1}$ is defined (see equation (37)) via the solution to the Dirichlet problem (38). By simple calculations, we obtain $N_{1}(\theta )\equiv -m\delta_{1}a$ , and the substitution of this value into equation (62) allows to determine the geometry perturbation parameter as:

(63) $$d_{\tau }=-\frac{\tau m\delta_{1}a^{2}}{N_{c}},$$

where it is assumed that $\delta_{1}<0$ which is the case in the process of decohesion.

The apparent non-uniqueness of the perturbation solution (63) (it should be remembered that the direction of the shift of the center of $\Gamma_{\tau }$ with respect to the center of Γ is arbitrary) is indicative of the instability of the decohesion process due to the axisymmetry of the initial contact configuration.

## 6. Discussion

First, it should be emphasized that the analysis presented above is based on the asymptotic model [7] of the JKR-type adhesive contact for a thin incompressible elastic layer. At the same time, the assumption of incompressibility is essential, because the layer’s deformation response becomes non-local compared to that of a thin compressible elastic layer, which responses to compression like a Winkler foundation (see, e.g., [12, 33]). It is of interest to note [34] that the model of an incompressible elastic material is applicable for describing the instantaneous response of a biphasic or poroelastic material [35, 36] (see also [12]). That is why, the developed asymptotic model has a wider area of application than just elastic incompressible coatings. We also note [37, 38] that the manifestation of adhesion is different from that of surface tension [39, 40]. Whereas the “inner” asymptotic model for a thin bonded incompressible elastic layer coated with an elastic membrane was derived in [41], the analysis of the boundary layer solutions, which allow to derive the boundary conditions on the contact contour, is still an open question.

Regarding the asymptotic character of equations (5)–(7), which assume that characteristic sizes of the contact domain ω are much larger than the thickness H of the elastic layer, it should be assumed that the maximum of the contact contour perturbation $h_{\tau }(s)$ , as introduced by equation (8), is larger than the layer thickness H . In other words, very small perturbations of the contact contour should be treated in the framework of the three-dimensional boundary-layer problem, e.g., using numerical methods [42].

Still, following Kolton and Nazarov [22], one can consider a localized asymptotics for the contact contour perturbation in terms of the fast coordinate $\xi \sim \tau^{-1/2}(s-s_{0})$ near a point $s=s_{0}$ of local maximum of the absolute value of the normal derivative of the limit solution $p_{0}(y)$ , i.e., $N_{0}(s_{0})=N_{c}$ . In this case, the second term on the left-hand side of the variational inequality (47) becomes dominant, but it can be shown that no *positive* solution (corresponding to the perturbation of the contact contour inside the initial contact domain) can exist for the resulting problem. By analogy with the asymptotic analysis of crack propagation [43], we conclude that the absence of solution or the existence of solution destroying the asymptotics (36) serves as an indication for the adhesive detachment in an instable way. This is well illustrated by one of the two examples considered above.

It would be tempting to deduce some mechanical conclusions from the considerations of instability of detachment process and non-uniqueness of solutions. However, it must be bared in mind that in the JKR type of adhesion, any viscous effects, which may depend on the rate of separation of the contacting surfaces, have been completely neglected. As such, the multiple solutions cannot be ranged with the account for the energy dissipated due to separation of the molecular bonds during the decohesion process.

When looking at the results of the displacement-driven decohesion experiments [4] through a prism of the constructed asymptotic model, we can make the following conclusions. The decohesion initiation at the interface between a flat-ended elliptical punch occurs near the vertices of the minor semi-axis of the initial elliptical area of contact. With increasing the eccentricity of the initial elliptical contact contour, the decohesion zone rapidly widens, as it follows from formula (54). However, further analytical studies are needed to extend the theory to the case of rate-dependent adhesion (see, e.g., [44]).
